# Salicylic Acid in Small-Pox

**Published:** 1876-11

**Authors:** 


SALICYLIC ACID IN SMALL-POX.—Dr. J. B. Trenley writes the editor of *Pacific Medical Journal* from Oakland, Cal:

DR. H. GIBBONS—*Dear Sir:* Through your courtesy, I am in receipt of the Sept. No. of the *Pacific Medical and Surgical Journal,* for which accept my thanks.

In it I noticed an article by H. Gibbons, M.D., on Small-Pox and Re-vaccination. The propositions therein set forth correspond so nearly with my experience in treating variola for many years, that I cannot do otherwise than congratulate you for your close observations, and also for putting them in such a succinct manner in print. In regard, however, to the different methods used to prevent pitting and scarring the skin, it seems to me that nearly all the recommendations as applications are worse than useless, and will most certainly increase the coalition of pustules, causing deeper ulceration and a greater accumulation of pus. The worst cases I ever saw of pitting were where collodion and gutta percha solution in chloroform were used.

The course I have pursued in the present epidemic in treating the disease, has been quite satisfactory, as regards disfigurement from pitting, and has been obtained in the use of *Salicylic Acid* both internally and externally. Those cases that have recovered from the disease scarcely show a vestige of pitting, notwithstanding their confluent character.

The medicine appears not only to exert the influence mentioned, but to a great extent holds a modifying power over the violence of the disease, and I feel that as a remedy it is well worth the attention of physicians; and if found by future observation to bear out my observation as far as I have used it, I shall feel amply paid in knowing that one therapeutic agent of value has been added to the catalogue of medicines in treating this disease."

